# Effects of Tobacco on the Eyes

**Published:** 1880-03

**Authors:** 


### EFFECTS OF TOBACCO ON THE EYES.

Dr. A. W. Calhoun, of Atlanta, in an essay on the poisonous action of tobacco (Trans. Med. Asso. of Georgia), refers to a woman (Mrs. F.), aged thirty, "who not only smokes her pipe freely, but is an immoderate 'dipper' of snuff. This has been a habit with her for six or seven years. The cloudy vision increasing towards night, the floating bodies, the white atrophic appearance of the optic disc and anemic retina are all present to a very marked degree, otherwise she is apparently very healthy. She was, with difficulty, persuaded to quit the use of the pipe and *brush*, and put upon strychnia and electricity. The recovery in the course of a few months, was perfect in one eye and partial in the other."

